# A Large Family of Antivirulence Regulators Modulates the Effects of Transcriptional Activators in Gram-negative Pathogenic Bacteria

**DOI:** 10.1371/journal.ppat.1004153

**Published:** 2014-05-29

**Authors:** Araceli E. Santiago, Fernando Ruiz-Perez, Noah Y. Jo, Vidhya Vijayakumar, Mei Q. Gong, James P. Nataro

**Affiliations:** 1 Department of Pediatrics, University of Virginia School of Medicine and University of Virginia Children's Hospital, Charlottesville, Virginia, United States of America; 2 Department of Microbiology and Immunology, University of Maryland School of Medicine, Baltimore, Maryland, United States of America; National Institute of Allergy and Infectious Diseases, National Institutes of Health, United States of America

## Abstract

We have reported that transcription of a hypothetical small open reading frame (orf60) in enteroaggregative *E. coli* (EAEC) strain 042 is impaired after mutation of *aggR*, which encodes a global virulence activator. We have also reported that the cryptic orf60 locus was linked to protection against EAEC diarrhea in two epidemiologic studies. Here, we report that the orf60 product acts as a negative regulator of *aggR* itself. The orf60 protein product lacks homology to known repressors, but displays 44–100% similarity to at least fifty previously undescribed small (<10 kDa) hypothetical proteins found in many gram negative pathogen genomes. Expression of orf60 homologs from enterotoxigenic *E. coli* (ETEC) repressed the expression of the AraC-transcriptional ETEC regulator CfaD/Rns and its regulon in ETEC strain H10407. Complementation *in trans* of EAEC 042*orf60* by orf60 homologs from ETEC and the mouse pathogen *Citrobacter rodentium* resulted in dramatic suppression of *aggR*. A *C. rodentium* orf60 homolog mutant showed increased levels of activator RegA and increased colonization of the adult mouse. We propose the name Aar (AggR-activated regulator) for the clinically and epidemiologically important orf60 product in EAEC, and postulate the existence of a large family of homologs among pathogenic *Enterobacteriaceae* and *Pasteurellaceae*. We propose the name ANR (AraC Negative Regulators) for this family.

## Author Summary

We report here the identification and characterization of a new family of negative regulators in Gram-negative bacteria, including many pathotypes of diarrheagenic *Enterobacteriaceae* and members of the *Pasteurellaceae*. Members of this regulator family in enteroaggregative (EAEC) and enterotoxigenic *E. coli* (ETEC) and in *Citrobacter rodentium* downregulate the expression of positive regulator partners AggR, CfaD/Rns and RegA, respectively, all members of the AraC/XylS family of regulators. Accordingly, we propose the name ANR (AraC Negative Regulators) for this family. ANR members orf60 (termed Aar), orf02851 (Rnr), orf0450 and orf01070 (Cnr) from EAEC, *C. rodentium* and ETEC respectively were characterized in this study. Deletion of ANR homologs upregulated the expression of AggR and RegA in EAEC strain 042 and *C. rodentium* respectively; overexpression of orf60, orf02851, orf0450 and orf01070 in EAEC strain 042 down-regulated AggR. *C. rodentium* harboring a null mutation in orf02851 exhibited a significant increase in expression of the *regA* and RegA-regulated fimbriae. The orf02851 mutant showed higher levels of *C. rodentium* in feces and colonic contents, and greater weight loss compared to mice inoculated with the wild-type.

## Introduction

Enteroaggregative *Escherichia coli* (EAEC) is a diarrheagenic pathotype linked to traveler's diarrhea, foodborne outbreaks and sporadic diarrhea in industrialized and developing countries [1]–[4]. A Shiga toxin-lysogenized EAEC caused a large and highly lethal European outbreak in 2011, highlighting the potential virulence of this pathotype [4]–[6].

Virulence gene expression in EAEC is activated in coordinate fashion by a regulator called AggR, a member of the AraC/XylS family of bacterial transcription factors [7]–[9]; AggR is most closely related to the CfaD/Rns activator of enterotoxigenic *E. coli* (ETEC) [10], RegA in *C. rodentium* [11] and PerA in enteropathogenic *E. coli* (EPEC) [12], [13]. AggR, Rns/CfaD, RegA and PerA are required for expression of genes mediating the biosynthesis of colonization fimbriae and other virulence-associated factors. We have recently reported that AggR regulates the expression of at least 44 genes, including itself, at the onset of the logarithmic growth phase [14].

Among the most dramatically upregulated genes in the AggR regulon was a very small cryptic open reading frame (orf; designated variously orf60 or orf61, but here only orf60) lacking homology to any known gene, but located on the pAA2 virulence plasmid nearby to the gene encoding AggR itself [14]. We were struck by the high prevalence and conservation of orf60 among EAEC strains. In an effort to understand the highly mosaic character of the EAEC genome and its relationship to endemic diarrhea in developing countries, we scored the presence of AggR-dependent and AggR-independent putative virulence genes among collections of EAEC strains from children under 5 years of age with diarrhea in Mali [15] and Brazil [16]. In both studies, we were struck by the strong and consistent negative association between the presence of orf60/61 and diarrhea. Given compelling data favoring some important role for cryptic locus orf60/61 in EAEC epidemiology, we undertook a thorough investigation of its role in pathogenesis.

We report here that EAEC orf60 encodes a novel protein negative regulator, now formally re-named Aar (AggR-activated regulator). Although expression of the Aar-encoding gene in EAEC is indeed activated by AggR, expression of the gene down-regulates expression of AggR itself. Most surprisingly, we show that Aar is a member of a previouly unrecognized large class of regulators in pathogenic Gram negative bacteria.

## Results

### Altered fimbrial expression in 042*orf60*

To examine the role of orf60, a 042*orf60* mutant was generated, assessed for established EAEC phenotypes and observed by TEM. The orf60 mutant was complemented *in trans* by expressing the predicted orf downstream of the arabinose promoter in pBAD30, to yield pOrf60. Since the orf generated two possible protein products given translational start from an ATG or alternative start codon, the construct included the most upstream (alternative) potential start site.

**Figure 1:**
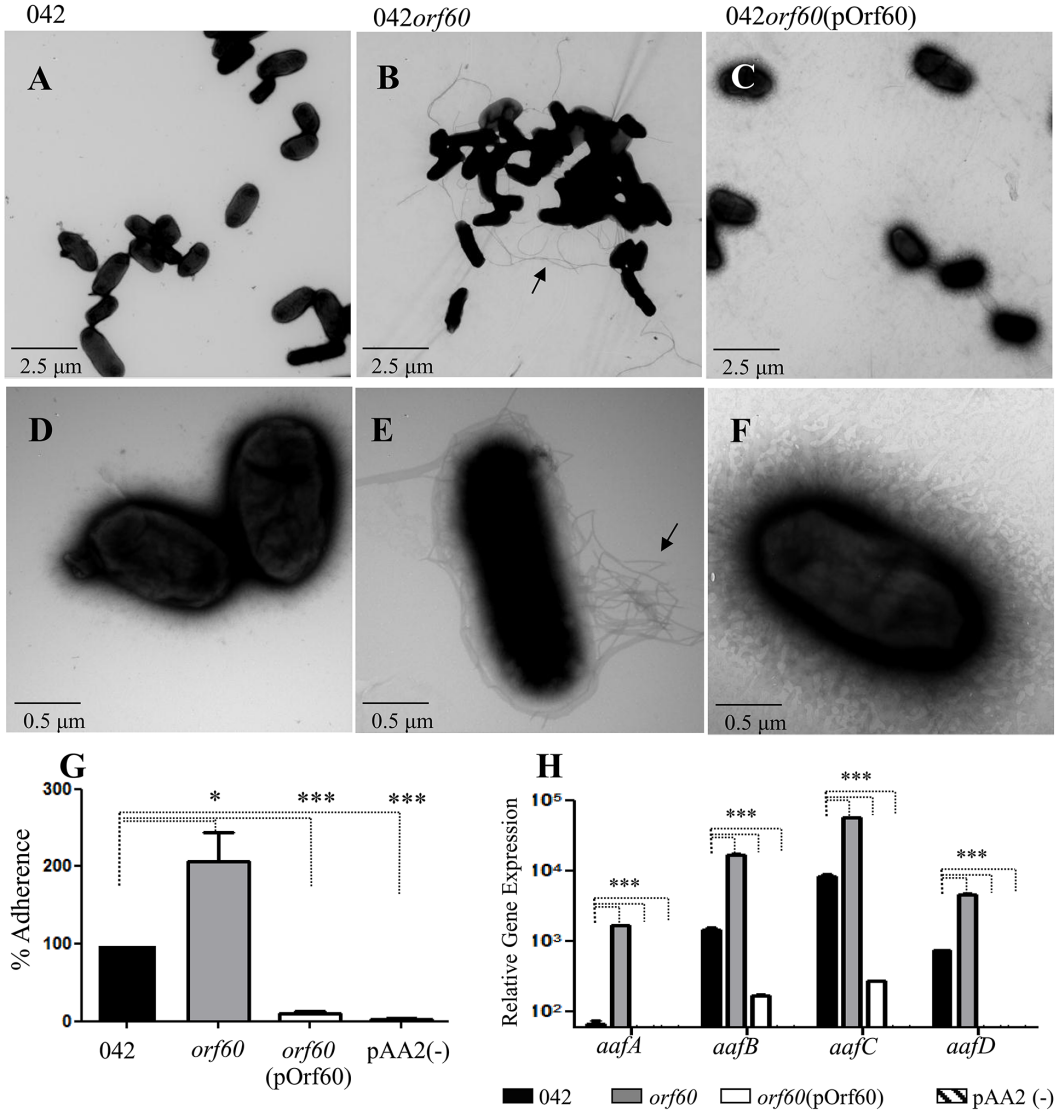
Increased expression of AAF in 042*orf60*. Transmission electron microscopy of negatively stained samples was performed for 042 (Panels A and D), 042*orf60* (Panels B and E), and 042*orf60*(pOrf60) (Panels C and F). Samples were visualized at a magnification of ×5,000 (Panels A, B and C), or ×25,000 (Panels D, E, and F). Bacterial adherence to fibronectin-coated plates was quantified as described in Materials and Methods (Panel G). Transcript levels for *aaf* fimbrial genes were measured in 042 (black bars), 042*orf60* (gray bars), 042*orf60*(pOrf60) (open bars) and 042pAA2(-) (negative control; striped bars) by qRT-PCR during the log phase of growth (Panel H). Asterisks indicate significant differences by ANOVA (***, P<0.0001; *, P<0.05).

Surprisingly, 042*orf60* was found to be hyper-fimbriated by negative staining in TEM (Figure 1, B and E), and readily distinguishable from the parent strain (Figure 1, A and D), or the 042*orf60* mutant complemented *in trans* with the pOrf60 plasmid (Figure 1, C and F), both of which showed sparse fimbriae. As predicted from the documented role of AAF in aggregation, larger bacterial aggregates were visualized by TEM in the 042*orf60* mutant, when compared to the wild type or complemented strains. The hyper-aggregative phenotype of 042*orf60* was also apparent in undisturbed broth cultures (Fig. S1 in Text S1, panels A and B). We have previously demonstrated that the AAF/II major pilin protein AafA binds to fibronectin [17]. To evaluate fimbrial function in the absence of orf60, we assessed this phenotype in 042*orf60* and complemented constructs. As expected, the hyperfimbriated 042*orf60* strain exhibited more abundant binding to fibronectin-coated plates (∼200% binding) compared to the parent strain (Figure 1, panel G), while overexpression of orf60 in the complemented EAEC strain yielded marked reduction of fibronectin binding.

As predicted from these phenotypes, 042*orf60* yielded more abundant expression of the major AAF/II fimbrial subunit protein AafA than its wild type parent in immunoblot analysis, and this phenotype was reversed in the complemented construct (Fig. S1 in Text S1, panel C). We therefore employed qRT-PCR to assess the effect of orf60 on *aaf* fimbrial gene expression. 042 wild type, 042*orf60* and 042*orf60*(pOrf60) strains were grown in DMEM high glucose and samples from log-phase cultures were used for qRT-PCR analysis (Figure 1, H). We observed higher levels of expression for fimbrial genes *aafA* (10- to 25-fold), *aafB* (4- to 11-fold), *aafC* (4- to 6-fold) and *aafD* (3- to 8-fold) in the 042*orf60* mutant compared with the parent strain, while frimbrial gene expression in the complemented strain was nearly undetectable.

### AggR and orf60 are cognate partners in a regulatory feedback system

**Figure 2:**
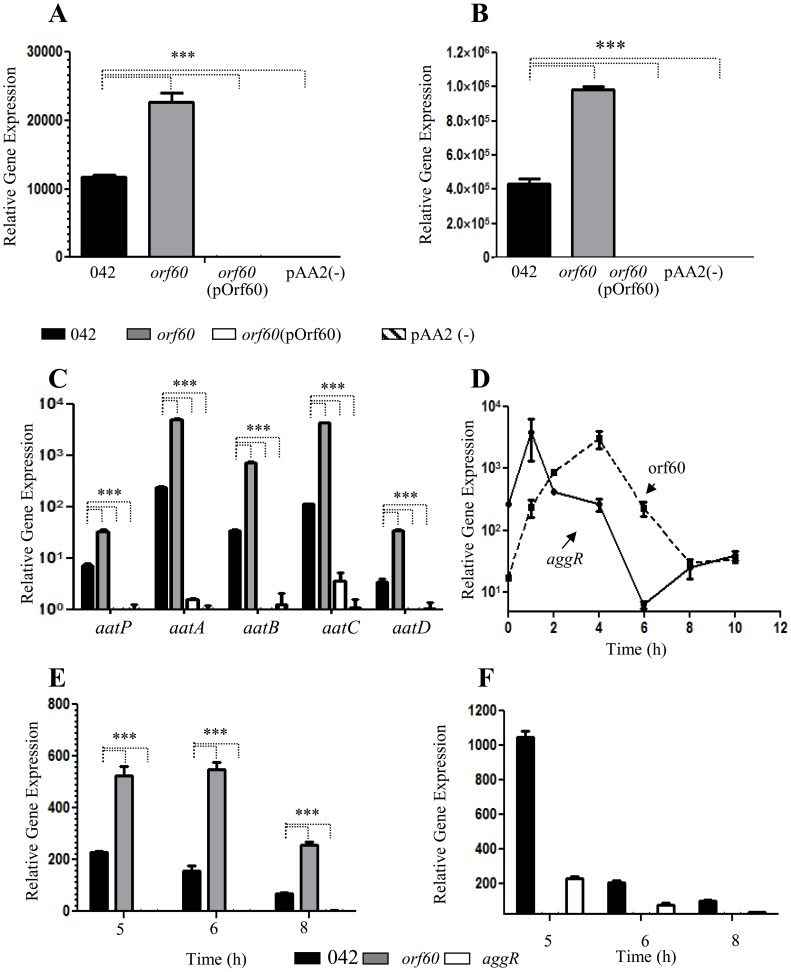
Orf60 represses expression of AggR and its regulon. Transcript levels for *aggR* (Panel A), *aap* (Panel B) and *aatABCDP* (Panel C) were evaluated in 042 (black bars), 042*orf60* (gray bars), 042*orf60*(pOrf60) (open bars) and 042pAA2(-) (striped bars) strains by qRT-PCR. Transcripts for *aggR* (Panel E) and orf60 (Panel F) were examined in the 042*aggR* and 042*orf60* mutant strains during the middle to the late log phase (5 to 8 h). Expression of *aggR* and orf60 was analyzed in the 042 wild type strain by qRT-PCR over time (Panel D). Asterisks indicate significant differences by ANOVA (***, P<0.0001).

Our data suggested that orf60 may act as a negative feedback regulator of fimbrial gene expression, despite the fact that both the fimbriae and orf60 are under AggR activation. We sought to dissect features of this regulatory system in EAEC using qRT-PCR. Levels of transcription of *aggR* (Figure 2, A) and the *aggR*-regulated genes *aap* (Figure 2, B) and *aatPABCD* (Figure 2, C) were compared between 042*orf60* and the parent strain by qRT-PCR. We observed two-fold higher *aggR* mRNA levels in 042*orf60* compared to the parent strain (Figure 2, A). Expression of other AggR-regulated genes was similarly affected by orf60 deletion, including *aap*, *aatP, aatA, aatB, aatC*, and *aatD* (Figure 2, B and C). The greatest effect was observed for the *aatC* gene, expression of which was 37-fold higher in the orf60 mutant compared with the wild type parent strain (Figure 2, C). Complementation *in trans* with the orf60 gene resulted in undetectable levels of *aggR* and AggR-regulated genes, comparable to the levels seen in EAEC lacking the pAA2 plasmid (Figure 2A, B and C). We examined similarly *aggR* and orf60 expression in the 042*aggR* and 042*orf60* mutant strains during the middle-late log phase of growth (5–8 h). *aggR* expression was consistently high in the absence of orf60 (Figure 2, E), whereas orf60 levels were drastically reduced in the absence of *aggR* (Figure 2, F). These data suggest that the effect of orf60 on fimbrial gene expression was via a negative feedback effect on *aggR* itself, accompanied by the predicted suppression of other genes downstream in the AggR regulon.

We hypothesized that orf60 may represent a delayed negative feedback regulator of *aggR*. To test this hypothesis, RNA was obtained from strain 042 cultures prepared in DMEM high glucose at various stages of cell growth, spanning the log and early stationary phases (0–8 h of growth), and analyzed for *aggR* and orf60 transcripts (Figure 2, D). As previously reported [18], we observed a rapid increase in *aggR* expression in the first 2 h of growth (early log phase), while expression of orf60 was delayed, achieving maximal levels after 4 h of incubation (middle log phase) (Figure 2, D); at this time point *aggR* expression was seen to be declining, consistent with activation of orf60 by AggR and repression of *aggR* by the orf60 product. At 6 h of growth, both *aggR* and orf60 levels were declining. Interestingly, orf60 gene expression reached its lowest expression level at the end of late log phase (8 h), while *aggR* expression was again increasing. Both *aggR* and orf60 were at low levels by 10 hr of growth (Figure 2, D).

### orf60 encodes a regulatory protein

**Figure 3:**
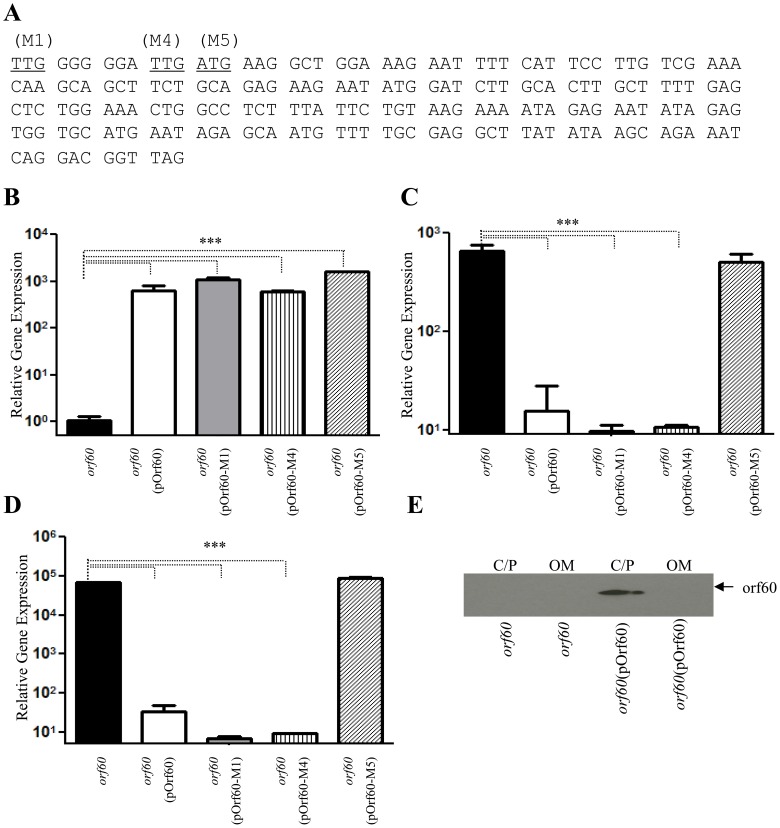
Orf60 encodes a regulatory protein. Hypothetical start codons TTG (M1 and M4) and ATG (M5) (Panel A), were substituted by stop codon TAG in the orf60 sequences of pOrf60. Plasmid derivatives (pOrf60-M1, pOrf60-M4, and pOrf60-M5) were used to complement the 042*orf60* strain. Transcripts for orf60 (panel B), *aggR* (panel C), and *aap* (panel D) were determined in all 042*orf60*(pOrf60) derivatives by qRT-PCR. Outer membrane (OM), and cytoplasmic/periplasmic fractions (C/P) of 042*orf60* and 042*orf60*(pOrf60) were extracted and analyzed by Western blot (Panel E). Asterisks indicate significant differences by ANOVA (***, P<0.0001).

We sought to determine whether orf60 encodes a small regulatory RNA molecule or a regulatory protein. Expression of orf60 from pOrf60 was assessed after mutation of three potential start codons in the open reading frame: predicted codons 1, 4 and 5 (illustrated in Figure 3A as M1, M4, and M5). Plasmid derivatives (pOrf60-M1, pOrf60-M4, and pOrf60-M5) were used to complement the 042*orf60* strain. Transcripts for orf60, *aggR* and *aap* (Figure 3, B, C and D) were determined in all 042*orf60*(pOrf60) derivatives. In all strains, high levels of transcripts for orf60 were detected by qRT-PCR (Figure 3, B). Replacement of the ATG only in position 5 with a stop codon lead to abrogation of the orf60-induced phenotypes, manifested as expression of *aggR* and the AggR-regulated gene *aap* (Figure 3, panels C, D). The hyper-aggregative phenotype of 042*orf60* was not reversed when the strain was transformed with pOrf60-M5, indicating requirement of this predicted start codon in orf60 expression (Fig. S1 in Text S1, panels A,B).

Our data suggested that orf60 would be transcribed and translated as a small protein of 7.23 kDa. In order to observe and localize the orf60 protein, bacterial samples were fractionated into outer membrane, cytoplasmic/periplasmic fractions, and analyzed by Western immunoblot (Figure 3, E). Localization of the product at the predicted mass confirmed its existence and localized the product to the cytoplasmic/periplasmic fraction of the bacterium.

Using the Bprom algorithm, a strong sigma-70 bacterial promoter region was predicted upstream of the ATG start site; predicted −35 and −10 regions were included in plasmid pOrf60. To confirm that constitutive expression of orf60 was driven by this predicted promoter, the *araC* gene and *P_BAD_* promoter were removed from pOrf60 plasmid to generate pOrf60-2 (Fig. S2 in Text S1, panel A). pOrf60-2 plasmid was transformed into 042 and its orf60 mutant, and RNA transcripts for orf60 and *aggR* were quantitated by qRT-PCR. High transcriptional levels of orf60 were detected by qRT-PCR in strains transformed with pOrf60 and pOrf60-2 (Fig. S2 in Text S1, panel C). 042 derivatives transformed with pOrf60-2 still retained the inhibitory effect of orf60 (Fig. S2 in Text S1, panel D).

High transcriptional levels of orf60 did not compromise the fitness of the cell, as manifested by growth curves in DMEM medium (Fig. S3 in Text S1, Panel A). To assure that the effects of orf60 expression in trans were not due to high copy number over-expression artifacts, we cloned the gene into low copy number vector a pACYC177. This lower copy number construct restored the wild type phenotype (Fig. S3 in Text S1, Panels B, C, D).

### orf60 belongs to a new family of regulators

**Figure 4:**
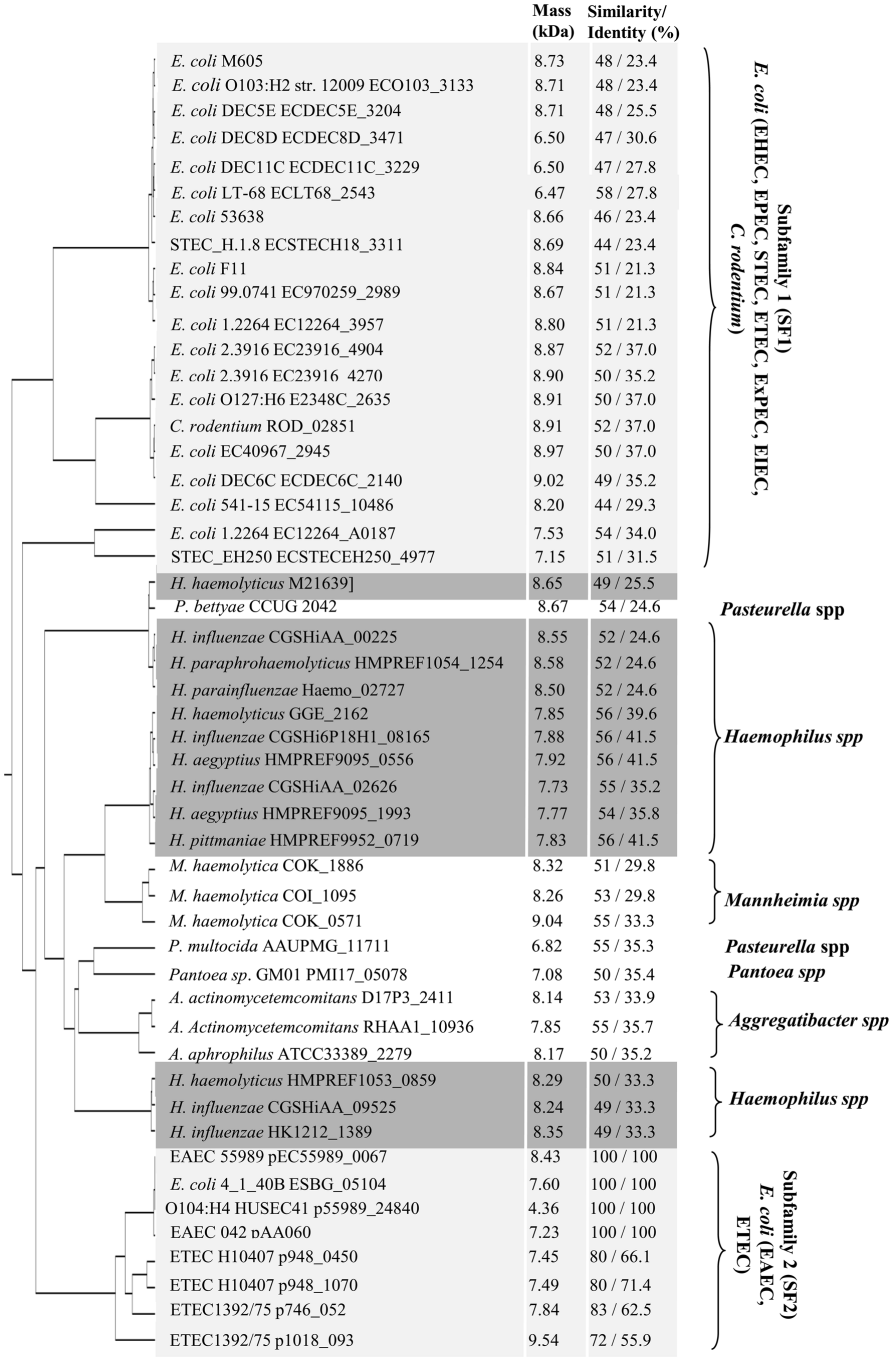
orf60, a new family of regulator proteins. Phylogenic analysis was performed for orf60 homologs by using the ClustalW algorithm. The protein molecular mass and percent identity and similarity to EAEC orf60 were determined for each predicted protein.

Blast analysis of orf60 and its predicted product did not suggest significant relatedness to any known bacterial regulatory protein; however, the analysis did identify nearly 50 hypothetical proteins, present in 7 major genera/species: *Escherichia coli*, *Citrobacter* spp., *Haemophilus* spp., *Pasteurella* spp., *Mannheimia* spp., *Pantoea* spp. and *Aggregatibacter* spp. (Figure 4). Like orf60, all predicted products of this family of hypothetical proteins exhibited low predicted molecular mass (39 to 80 amino acids, 4.36–9.54 kDa), and exhibited 44–100% similarity to orf60 (Figure 4).

*Haemophilus* orf60 homologs were mainly found in pathogenic strains including *H. influenzae, H. paraphrohaemolyticus, H. parainfluenzae, H. aegypticus*, and *H. pittmaniae* subspecies [19], [20]. The predicted protein masses of orf60 homologs in *Haemophilus* sp. varied from 7.73 to 8.65 kDa and exhibited 49 to 56% amino acid similarity to the EAEC orf60 product (Figure 4, dark gray boxes). orf60 homologs were also found in pathogens *Pasteurella multocida* and *Pasteurella bettyae* [21]; these predicted proteins displayed 54% similarity to orf60 with sizes between 6.82 to 8.67 kDa (Figure 4). Orf60 homologs with 51 to 55% similarity and masses between 8.26 to 9.04 kDa were also identified in *Mannheimia haemolytica*, the agent of bovine respiratory disease complex [22].

Orf60 homologs in *Aggregatibacter actinomycetemcomitans* and *Aggregatibacter aphrophilus* exhibit 50 to 55% similarity to EAEC orf60 and their masses range from of 7.85 to 8.17 kDa. Orf60 homologs are also found in *Pantoea* spp., a Gram-negative bacterium associated with infections in neonates [23]. The orf60 homolog in *Pantoea* is a 7 kDa protein that shows 50% similarity to EAEC orf60.

### orf60 homologs of pathogenic *E. coli* strains

A phylogenetic subgroup of orf60 homologs was found among pathogenic *E. coli* genomes, subdivided into two main subfamilies (SFs), SF1 and SF2 (Figure 4). SF1 comprised the orf60 homologs found in enterohemorrhagic *E. coli* (EHEC) strain (12009, DEC5E, DEC8D), enteropathogenic *E. coli* (EPEC) (E2348/69), Shiga-toxin producing *E. coli* (STEC) (DEC11C, H.1.8, EH250, 99.0741, 1.2264, 2.3916), enterotoxigenic *E. coli* (ETEC) (LT-68), extra-intestinal pathogenic *E. coli* (ExPEC) (F11), enteroinvasive *E. coli* (EIEC) (53638); homologs were identified among additional *E. coli* strains designated M605, EC40967, DEC6c and 541-15. Orf60 homologs in the SF1 subfamily share 44 to 58% amino acid identity across the full length of the predicted proteins (Figure 4, light gray box). SF2 comprised homologs among EAEC (55989), ETEC (H10407, and 1392/75), and Shiga-toxin producing EAEC (HUSE41) strains; the archetype orf60 from 042 was assigned to this SF, all members of which shared 72–100% amino acid similarity (Figure 4).

Among the most noteworthy predicted proteins was ROD_02851 (8.9 kDa) from *C. rodentium*, sharing 52% similarity to orf60 (Figure 4). *C. rodentium* is an enteric pathogen which pursues a pathogenic strategy similar to that of EPEC and STEC, and which harbors both an AggR homolog (RegA) and an orf60 homolog.

**Figure 5:**
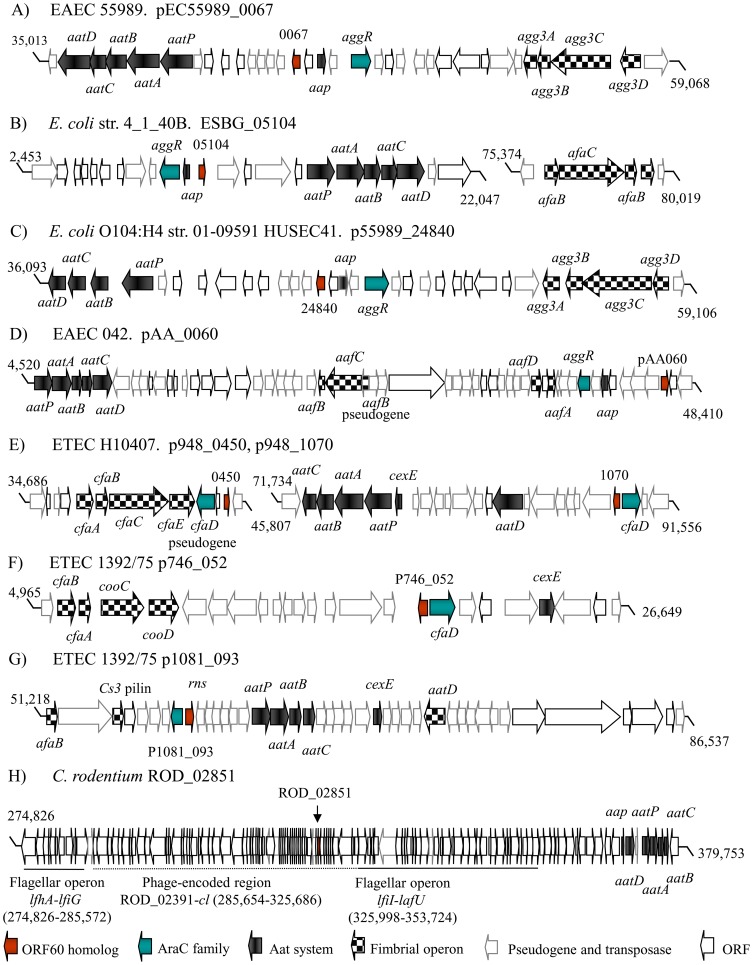
Genetic organization of orf60 homologs. DNA sequences for EAEC 55989 (35,013–59,068; GenBank NC_011752.1) (Panel A), *E. coli* str. 4_1_40B (2,453–22,047, 75,374–80,019; GenBank NZ_JH660546.1) (Panel B), *E. coli* O104:H4 str. 01-09591 HUSEC41 (36,093–59,106; GenBank NZ_AFPS01000102.1) (Panel C), EAEC 042 (4,520–48,410; GenBank FN554767.1) (Panel D), ETEC H10407 (34,686–45,807, 71,734–91,556; GenBank NC_017724.1) (Panel E), ETEC 1392/75 (4,965–26,649; GenBank NC_014234.1 and 51,218–86,537; GenBank NC_014232.1) (Panel F and G) and *C. rodentium* (274,826–379,753; GenBank NC_013716.1) (Panel H) were examined. Orf60 alleles (red arrows), AraC/XylS homologs (blue arrows), fimbrial operons (checkered arrows), and dispersin/Aat translocator systems (black arrows) are indicated.

We examined the genetic organization of the orf60 homologs among the diarrheagenic *E. coli* strains EAEC, ETEC, Shiga-toxin producing EAEC and *C. rodentium*. Unexpectively, in all the analyzed EAEC and ETEC genome sequences, four highly conserved features were found: a fimbrial operon, a dispersin-like protein, an Aat translocator system for dispersin, and an AraC family transcriptional regulator. Whereas the precise location of the genes varied, all were found to be located close to and in divergent organization up or downstream of AraC activators (Figure 5, A–G).

Most interesting was the case of archetype ETEC strain H10407 [24]. This strain has been described as having two copies of *cfaD/rns*, which encodes the AraC homolog most closely related to AggR; one of the copies exhibits a frameshift mutation and is therefore considered to be inactive [25]. We found orf60 homologs divergently arranged in close proximity to both *cfaD* genes (Figure 5, E). Another ETEC strain, 1392/75, harbors two orf60 genes, located in the p746 (p746_052) and p1018 (p1018_093) plasmids (Figure 5, F, G). In contrast to EAEC and ETEC, the orf60 homolog of *C. rodentium* was located within a predicted phage-encoded region, flanked by flagellar operon genes and the Aat translocator system (Figure 5, H).

Conservation of function would presumably dictate conservation of orf60 protein structure across the family. To test this hypothesis, we assembled an alignment of orf60 family members and applied a variety of prediction algorithms to reveal the presence of conserved predicted secondary structure. PROMALS3D algorithm strongly predicted the presence of three consensus alpha helices at conserved locations in the family (Fig. S4). Several amino acid residues were highly conserved in identity or character across the family; these included a very highly conserved cysteine at position 60 in the EAEC orf60 protein.

### orf60 homologs trans-complement 042*orf60* phenotype

We sought to address whether orf60 homologs from other diarrheagenic strains would be able to rescue orf60 function in the 042*orf60* mutant. For these experiments we chose orf60 homologs from SF1 (*C. rodentium*, ROD_02851) and SF2 (ETEC strain H10407, p948_0450 and p948_1070), exhibiting similarities of 52 and 80% to orf60 respectively (Fig. 4).

**Figure 6:**
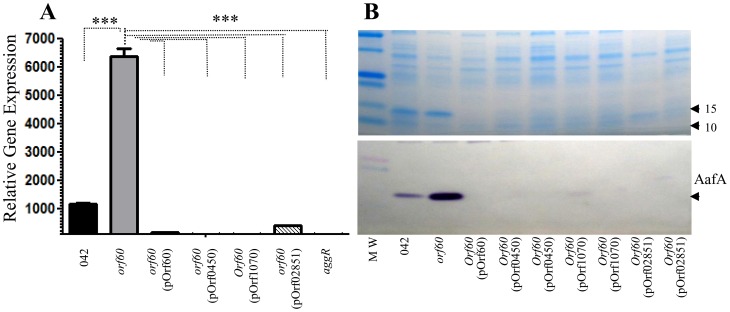
orf60 homologs from ETEC and *C. rodentium* repress AggR. 042*orf60* was complemented *in trans* with orf60 homologs from ETEC (p948_0450 and p948_1070) and *C. rodentium* (ROD_02851). Expression of AafA was evaluated by SDS-PAGE and Western blot (Panel B) using anti-AafA antibody. Transcripts for *aggR* were evaluated in the strains by qRT-PCR (Panel A). Asterisks indicate significant differences by ANOVA (***, P<0.0001).

We transformed 042*orf60* with pOrf0450, pOrf1070, and pOrf02851; strains were grown in DMEM high glucose, then proteins and RNA were analyzed by western blot using anti-AafA antibody and qRT-PCR, respectively. AafA protein expression in 042 was completely abolished when complemented with any of the orf60 homologs (Fig. 6B). Similarly, *aggR* expression was suppressed in strains complemented with any of the ETEC or *C. rodentium* orf60 homologs (Fig. 6A). Thus, orf60 homologs from two different subfamilies were capable of complementing orf60 function in 042.

### ETEC orf60 homologs repress CfaD/Rns and its regulon

**Figure 7:**
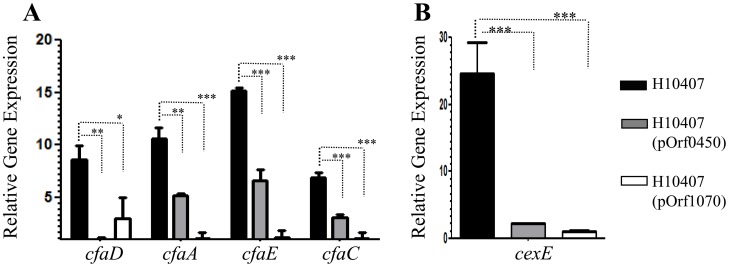
Repression of *cfaD/rns* and its regulon by ETEC-orf60 homologs. Transcripts for *cfaD/rns*, its fimbrial operon (*cfaA, cfaE*, and *cfaC*) (Panel A), and dispersin-like *cexE* (Panel B) were evaluated in ETEC H10407 (black bars), H10407(pOrf0450) (gray bars) and H10407(pOrf1070) (open bars) strains by qRT-PCR. Asterisks indicate significant differences by ANOVA (***, P<0.0001; **, P<0.001; *, P<0.05).

We hypothesized that the orf60 homologs from ETEC acted as regulators of the AggR homolog Rns/CfaD. ETEC H10407 was transformed with pOrf0450 and pOrf1070, grown and prepared for qRT-PCR analysis as above. In the presence of the orf60 homologs, we observed significant reduction in expression of *cfaD* itself (3- to 8-fold), as well as the CfaD-activated fimbrial genes, *cfaA* (2- to 9-fold), *cfaE* (2- to 13-fold), *cfaC* (4- to 12-fold) genes, and the gene encoding the dispersin-like protein CexE (11- to 24-fold) (Fig. 7, A, B). Interestingly, the orf1070 gene repressed *cfaD* and CfaD-regulated genes more strongly than did orf0450.

### *C. rodentium* orf60 homolog repress RegA and its regulon

**Figure 8:**
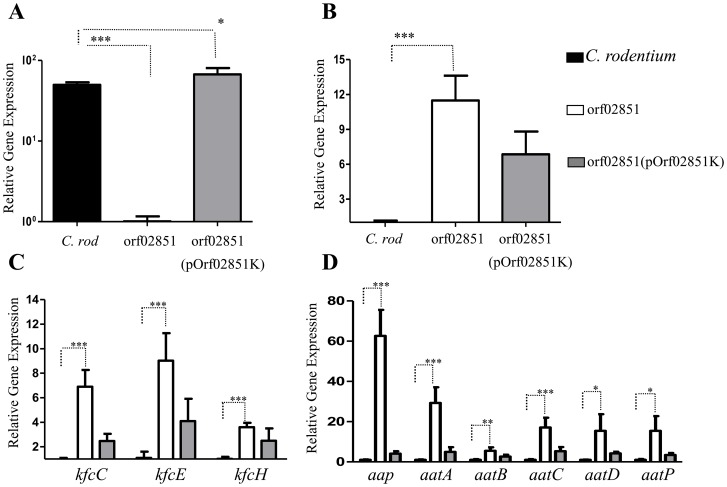
Repression of *regA* and its regulon by orf60 homolog-orf02851. Transcripts for orf02851 (Panel A), *regA* (panel B), fimbrial operon encoding genes (*kfcC, kfcE*, and *kfcH*) (Panel C), dispersin-like gene and *aat* system (Panel D) were evaluated in *C. rodentium* (black bars), orf02851 mutant (open bars) and orf02851(pOrf02851K) (gray bars) strains by qRT-PCR. Asterisks indicate significant differences by ANOVA (***, P<0.0001; **, P<0.001; *, P<0.05).

We sought to determine if the orf60 homolog of *C. rodentium* (ROD_02851) is a negative regulator of the AggR homolog RegA (Fig. 8). *C. rodentium* was transformed with pOrf02851K, grown and prepared for qRT-PCR analysis as above. In the absence of orf02851, we observed a significant increase in expression of *regA* (∼10- to 12-fold) (Fig. 8, B), as well as the genes regulated by RegA: fimbrial genes *kfcC, kfcE* and *kfcH* (4- to 11-fold) (Fig. 8, C), dispersin (∼80-fold) and the *aat* system (20- to 30-fold) (Fig. 8, D). Complementation *in trans* down-regulated the expression of *regA* and RegA-regulated genes (Fig. 8, B, C, D). Of note, mutations in the Kfc pilus genes were found to demonstrate early loss of colonization by *C. rodentium* [11].

### Virulence of the *C. rodentium* orf02851 mutant in the C57BL/6 mouse model

**Figure 9:**
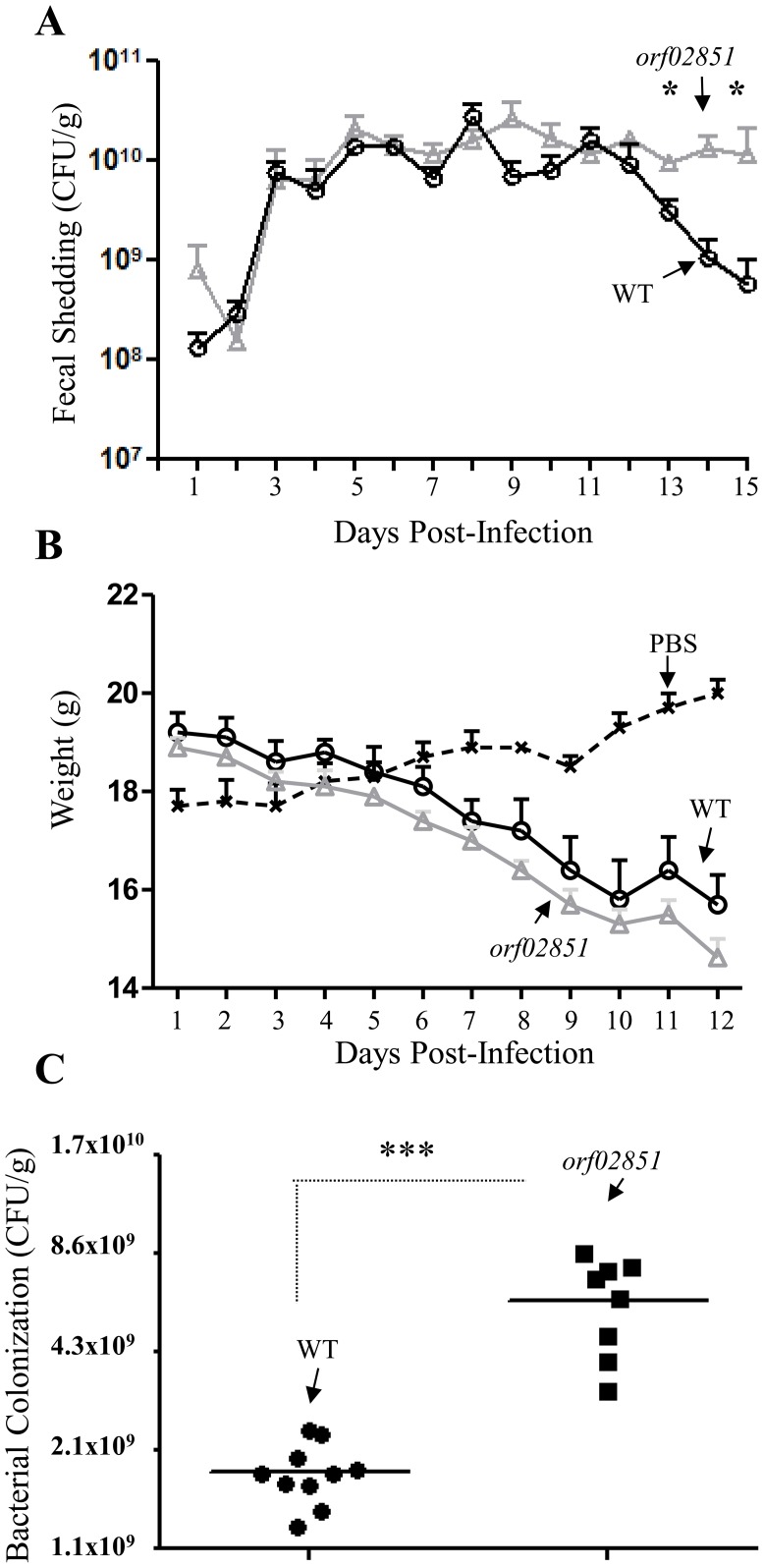
Colonization of C57BL/6 mice with derivatives of *C. rodentium*. Bacterial shedding was quantified in 5 mice at selected time points after inoculations (Panel A). Mice received orally 10^10^ CFU of *C. rodentium* (O) and orf02851 mutant (Δ) strains. As negative control 5 mice were inoculated with PBS (x). The mice were weighed daily and weigh loss was determined for each group (Panel B). Bacterial colonization in intestinal tissue was quantified in mice at day 9 post-inoculation (Panel C). Asterisks indicate significant differences by ANOVA (*, P<0.05).

**Figure 10:**
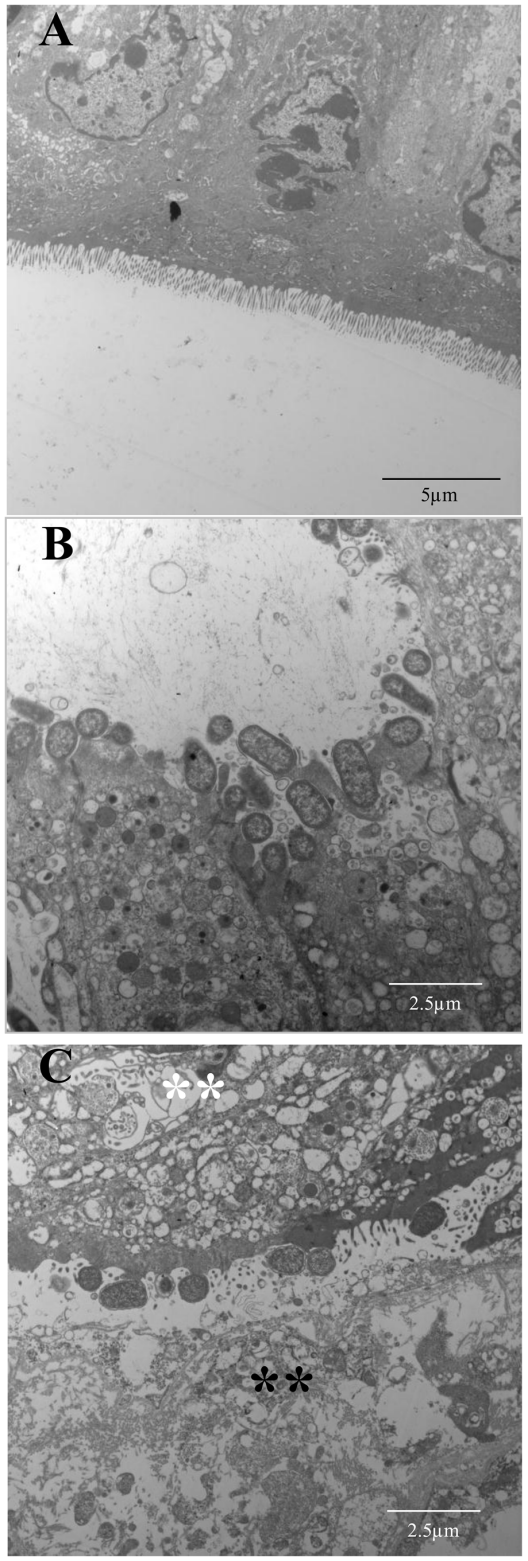
Transmission Electron Microscopy of the intestinal tissues of mice infected with *C. rodentium* or its orf02851 mutant. Groups of 5 mice were inoculated orally with 10^10^ CFU of *C. rodentium* (Panel B) and orf02851 mutant (Panel C) strains. Five mice were inoculated with PBS as control (Panel A). Mice were euthanized on day 9 and colonic tissues were prepared for TEM analysis. White and black asterisks indicate cellular vacuolization and debris observed in orf02851 mutant respectively (Panel C). Samples were visualized at a magnification of ×3,000 (Panel A), or ×5,000 (Panels B and C).

To examine potential effects of orf60-homolog orf02851 on virulence, groups of five mice were inoculated with the wild-type *C. rodentium* strain and the orf02851 mutant. Although both strains were recovered at high levels in stools collected from days 1 to 15 (Figure 9A), orf02851 mutant showed sustained high levels of bacteria (10^7^ to 10^10^) for more than 11 days (day 3 to 15) (Figure 9, A), whereas the wild-type strain exhibited a significant decrease in bacterial shedding levels after day 12 (Fig. 9, A). Only two mice out of five were shedding the wild type strain by day 16 and 17 (Table S3 in Text S2), while high levels of bacteria were detected in feces of mice inoculated with the orf02851 mutant (at levels of 10^7^ to 10^8^ per gram of stool) (Table S3 in Text S2). Consistent with levels of shedding in the stools, we observed higher levels of challenge strain colonization in the colonic lumen in mice fed the wild type strain compared with those fed the orf02851 mutant (Fig. 9C). Mice inoculated with *orf02851* strain also showed greater weight loss compared to mice inoculated with wild-type (Fig. 9B). Transmission electron microscopic examination of colonic tissues revealed more intense cytoplasmic vacuolization in animals infected with the mutant strain compared with wild type, accompanied by greater amounts of luminal debris (Fig. 10).

## Discussion

Many microorganisms utilize members of large families of regulators, presumably tailoring them to their pathogenetic idiosyncrasies. The large AraC/XylS family of gram-negative transcriptional activators is defined by a 100-amino-acid region of sequence similarity that contains two helix-turn-helix (HTH) DNA binding motifs [7], [9]; a number of family members are involved in carbon metabolism, responses to environmental stress, and many serve as virulence gene regulators [7], [9]. Several virulence-related AraC-family activators, including AggR of EAEC, have been shown to exercise autoactivation, presumably in order to assure very rapid activation of their regulons upon entry into an environment conducive to pathogenic lifestyles. Interestingly, however, for few autoactivators has a very simple engineering question been plausibly addressed: how does an autoactivator check runaway positive feedback? Unrestrained activation could be maladaptive in terms of optimal bacterial metabolism, or could conceivably render the bacterium more virulent than is fitting for optimal persistence in the host population. For these reasons, mutations in negative feedback regulators of autoactivators could engender increased or decreased virulence.

We suggest here the term Aar (AggR-activated regulator) to describe the orf60 gene product, and propose the term AraC-family negative regulators (ANR) to describe its family. The precise contribution of Aar in pathogenesis remains unknown. It could act by simply preventing excessive action of its activator partner, and/or could assure finer levels of on-demand gene expression at precise times or places in the setting of pathogenesis. Alternatively, Aar could act directly or indirectly as a virulence suppressor, perhaps to modulate virulence as a result of selection towards clinical attenuation. Our finding of increased pathogenicity in *aar*-negative strains in Mali and Brazil would support this latter hypothesis [15], [16]. Although the Aar-homolog mutation in *C. rodentium* yielded a predicted effect (enhanced intestinal colonization), it is unclear whether all Aar homologs act as virulence modulators.

The mechanism by which orf60 checks *aggR* expression is not apparent from our analysis. Alignment of the orf60 family revealed conserved motifs: strongly predicted alpha helical structure with several highly conserved residues (Figure S4). Alpha-helical dominance is found in several DNA binding proteins [26], suggesting that this function could be provided by orf60. Alternatively, orf60 could bind AggR, preventing its interaction with the DNA upstream of its activated promoters. A third possibility entails action of orf60 via an intermediate regulator. These three, and other possible hypotheses are under investigation.

The existence of antivirulence genes is attracting increased attention. Early work on this area suggested the loss of basic metabolic genes whose activities interfered with virulence functions (e.g. *cadA* of *Shigella* spp.) [27]. Some pathogens express protein effectors that act to suppress virulence, often by diminishing the inflammatory response (e.g. Pic from *Shigella flexneri*) [28]. However, to our knowledge, Aar represents the first example of a dedicated pathogen-specific attenuator of virulence genes in gram-negative bacteria. Additional investigations are required to understand the role of Aar and its homologs in the setting of disease.

## Materials and Methods

### Bacterial strain and growth conditions

Bacterial strains used in this study are shown in table S1 in Text S2. Strains EAEC 042, 042*aggR*, and 042*aggR*(pBAD*aggR*) were previously described [18], [29], [30]. Bacterial cultures were routinely propagated in Luria Broth (LB) and Dulbecco's modified Eagle's medium with 0.4% glucose (DMEM high glucose) (Gibco, Grand Island, NY) as previously described [14]. Mutagenesis of orf60 in 042 and *C. rodentium* strain ROD_02851 was accomplished by using lambda red technology [31]. The locus (46,236–46,438, GenBank FN554767.1) in 042 and (319,732–319,967, GenBank NC_013716.1) in *C. rodentium* were replaced with the kanamycin (km) resistance marker as previously reported [31]. 042*orf60* and *C. rodentium orf02851* strains were identified by PCR using specific primers for orf60, orf02851 and a km resistance marker (Table S2 in Text S2). We isolated a spontaneous pAA2 plasmid-cured derivative of strain 042 for use as a negative control in several assays.

### Complementation of 042*orf60* and *C. rodentium orf02851*

For the complementation of 042*orf60*, plasmids pOrf60, pOrf0450, pOrf1070 and pOrf2851 were generated in this study (Table S1 in Text S2). Briefly, a 328-kb fragment encompassing the orf60 gene and its predicted promoter (see Fig. S2 in Text S1) was amplified by PCR (spanning nucleotides 46,141–46,433 in GenBank accession FN554767.1), and cloned into the *Eco*RI and *Xba*I sites of pBAD30; the resulting plasmid was designated pOrf60. pOrf60-2 was generated by removing *araC* and the *P_BAD_* promoter from the plasmid vector backbone (Fig. S2 in Text S1). The orf60 gene with native promoter was generated by PCR (pACYC primers) (Table S2 in Text S2) and cloned into the *Hind*III/*Sma*I site of vector pACYC177 (GenBank X06402) (Figure. S3 in Text S1).

To express orf60 alleles from other bacteria, the EAEC orf60 gene was deleted by reverse PCR of pOrf60 so as to preserve the original EAEC orf60 translational start codon; the respective heterologous allele was fused downstream to assure transcription and translation at levels similar to EAEC. orf60 homologs from ETEC (p948_0450, genomic region 43,990–44,184 and p948_1070, genomic region 88,912–89,109, GenBank NC_017724.1) [32] and *C. rodentium* (ROD_02851, genomic region c319966–319733, GenBank NC_013716.1) [33] were employed to generate plasmids pOrf0450, pOrf1070, and pOrf2851 respectively. For the complementation of *C. rodentium orf02851*, the pOrf2851K plasmid was generated by inserting an encoding km^r^ marker into pOrf2851 backbone.

### Site directed mutagenesis of orf60 start codons

Stop codons (TAG) were introduced in the three hypothetical alternative or canonical start codons for orf60 ((TTG-position 1), 46,234–46,236; (TTG-position 4), 46,243–46,248 and (ATG-position 5), 46,246–46,248, (GenBank FN554767.1)) by using the QuikChange II XL Site-Directed Mutagenesis Kit (Agilent Technologies, CA USA). As a template for the mutagenesis reaction, 50 ng of pOrf60 plasmid was mixed with 125 ng of the corresponding primers (L1F-L1R; L4F-L4R and M5F-M5R) (Table S2 in Text S2) and 2.5 U/µl of Pfu Turbo DNA polymerase. Samples were treated for 1 cycle/3 minutes at 95°C followed by 18 cycles of 95°C/30 seconds, 55°C/1 minute, 68°C/8 minutes. Samples were digested with *Dpn*I for 2 h at 37°C and transformed into XL10-Gold Ultracompetent Cells (Agilent Technologies, CA USA). Plasmid derivatives were designated pOrf60-M1, pOrf60-M4, and pOrf60-M5 respectively. All constructs were verified by nucleotide sequencing at the University of Virginia DNA Science Core.

### Real-time quantitative reverse transcription-PCR (qRT-PCR)

Overnight bacterial cultures of EAEC were diluted 1∶100 into 13 ml of DMEM high glucose (*aggR*-inducing conditions), and incubated at 37°C without shaking for the times indicated. For ETEC and *C. rodentium*, bacterial cultures were diluted 1∶100 in 13 ml of LB or DMEM-LB broth respectively. Extraction of RNA, cDNA synthesis and qRT-PCR assays were performed as previously described [14]. Primers for EAEC were previously published [14]. Primers for ETEC and *C. rodentium* genes are reported in Table S2 in Text S2. Reactions were run in experimental duplicate using two independent cDNA preparations. Expression levels for each queried gene were normalized to the constitutively expressed *cat* gene in EAEC 042, *rpoA* in ETEC H10407 and *rpoD* in *C. rodentium* as previously described [14], [34], [35].

### Transmission Electron Microscopy (TEM)

Strains were grown in LB overnight with shaking at 37°C, then diluted 1∶100 in 5.0 ml of DMEM-high glucose, and incubated with shaking to reach OD_600_ of 0.8. Samples were prepared for negative staining with 2% uranyl acetate as previously described [14]. Colonic sections of mice infected with *C. rodentium* derivatives were analyzed by TEM. Briefly, groups of five C57BL/6 mice were inoculated with 10^10^ CFU of *C. rodentium* derivatives. Mice were euthanized at day 9 post-infection and descending colonic tissue was dissected and prepared for TEM analysis. The TEM samples were viewed on a Jeol JEM1230 Transmission Electron Microscope (80 kV) at the Advanced Microscopy Laboratory at the University of Virginia (AML-UVA).

### Detection of AAF/II and orf60 proteins

For detection of EAEC Aggregative Adherence Fimbriae II (AAF/II), strains were grown in 13 ml of DMEM high glucose to reach an OD_600_ of 0.8. Bacteria were pelleted, resuspended in 100 µl of 0.5 mM Tris, 75 mM NaCl and heated for 30 min at 65°C. The major pilin subunit of AAF/II (AafA) was analyzed in the supernatant by SDS-PAGE and Western blot analysis. For detection of orf60 protein, outer membrane (OM) and cytoplasmic/periplasmic (C/P) fractions of *E. coli* 042*orf60* and 042*orf60*(pOrf60) were prepared as described [36]. Protein samples were separated in acrylamide gels and transferred to Immobilon-P membranes (BioRad, Hercules CA, USA) by using standard protocols. The membranes were incubated overnight with anti-AafA or anti-orf60 antibodies respectively. The next day, the membranes were washed twice in PBS-0.1% tween, and incubated for 1 h with a horseradish peroxidase-conjugated goat anti-rabbit IgG antibody. Membranes were developed by using TMB Membrane peroxidase substrate (KPL, Gaithersburg, MD, USA) following the manufacter's specifications.

### AAF phenotype assays

Fibronectin-binding was assessed as previously reported [17]. The autoaggregation assay was performed as previously described (13).

### Bioinformatic and statistical analysis

Hypothetical proteins were analyzed by using Clustalw algorithms (http://www.genome.jp/tools/clustalw/). Protein mass, secondary structure and similarity was determined in predicted proteins by using bioinformatic tools (http://www.bioinformatics.org/sms/prot_mw.html, http://www.ch.embnet.org/software/LALIGN_form.html and http://prodata.swmed.edu/promals3d/promals3d.php. −35 and −10 regions of the *P_orf60_* promoter were predicted by using Bprom algorithm (http://linux1.softberry.com/cgi-bin/programs/gfindb/bprom.pl). Statistical analysis of the data for fibronectin binding, fecal shedding and gene expression was performed by using the GraphPad Prism 6 (GraphPad Software, Inc., CA, USA). The statistical significance of the differences in the sample means was calculated by using ANOVA with post hoc Tukey's correction. Results were considered significant at P<0.05.

### *C. rodentium* mouse model

All animal work has been conducted according to relevant national and international guidelines. Four to five week old male C57BL/6 mice were inoculated with 200 µl of a bacterial suspension containing 10^10^ CFU in PBS using a feeding needle. The mice were weighed daily and fecal pellets were collected aseptically from each mouse. The number of viable bacteria per gram of feces was determined by plating serial dilutions of the samples onto media containing appropriate antibiotics. For bacterial colonization, groups of 8–10 mice were inoculated with containing 10^10^ CFU in 200 µl PBS. Mice were euthanized at day 9 post-infection and bacteria in the large intestinal lumen was quantified as described above.

### Ethics statement

Animal experiments were performed in accordance with the Guide for the Care and Use of Laboratory Animals of the National Institutes of Health and with the permission of the American Association for the Assessment and Accreditation of Laboratory Animal Care. The protocol was reviewed and approved by the Institutional Animal Care and Use Committee of the University of Virginia (Protocol No. 3894).

### Accession numbers

Presented are database accession numbers for genes identified by BLAST search and presented in Fig. 4. Numbers are listed in order of similarity to orf60 from strain 042: gi|387604910, YP_006099179.1; gi|218511215,|YP_002415673.1; gi|386283102, ZP_10060736.1; gi|387610422, YP_006203859.1; gi|387610470, YP_006203907.1; gi|299836136, YP_003717705.1; gi|417835927, ZP_12482356.1; gi|298206490, YP_003717592.1; gi|419923404, ZP_14441355.1; gi|417147810, ZP_11988310.1; gi|215487703, YP_002330134.1; gi|283784065, YP_003363930.1; gi|417264971, ZP_12052353.1; gi|419153984, ZP_13698552.1; gi|417251191, ZP_12042956.1; gi|417259639, ZP_12047171.1; gi|191174000, ZP_03035517.1; gi|419137699, ZP_13682492.1; gi|417150722, ZP_11990461.1; gi|331648301, ZP_08349390.1; gi|417166561, ZP_11999917.1; gi|260845240, YP_003223018.1; gi|419216701, ZP_13759700.1; gi|417624551, ZP_12274849.1; gi|188493508, ZP_03000778.1; gi|419301338, ZP_13843337.1; gi|415811870, ZP_11504183.1; gi|417615862, ZP_12266306.1; gi|270315363, gb|EFA27649.1; gi|338217140, gb|EGP03044.1; gi|341952232, gb|EGT78764.1; gi|341956103, gb|EGT82542.1; gi|347813358, gb|EGY30032.1; gi|348653724, gb|EGY69408.1; gi|359755683, gb|EHK89847.1; gi|386908035, gb|EIJ72734.1; gi|145634275, ZP_01789986.1; gi|145635267, ZP_01790971.1; gi|145635897, ZP_01791585.1; gi|229844281, ZP_04464421.1; gi|261492133, ZP_05988704.1; gi|261493480, ZP_05990003.1; gi|261495320, ZP_05991771.1; gi|329122764, ZP_08251338.1; gi|329124231, ZP_08252775.1; gi|343518941, ZP_08755927.1; gi|359299044, ZP_09184883.1; gi|386389116, ZP_10073947.1; gi|387770888, ZP_10127061.1; gi|398801995, ZP_10561225.1

## Supporting Information

Text S1 **Supporting figures.** This file contains Figures S1–S4.
(PPT)

Text S2 **Supporting tables.** This file contains Tables S1–S3.
(DOC)
