# Supplementary material for: A Large Family of Antivirulence Regulators Modulates the Effects of Transcriptional Activators in Gram-negative Pathogenic Bacteria
Source: PLoS Pathog. 2014 May 29;10(5):e1004153. doi: 10.1371/journal.ppat.1004153 (PMC4038620; doi:10.1371/journal.ppat.1004153)
Supplement: Text S1 — Supporting figures. This file contains Figures S1–S4. (PPT) [file ppat.1004153.s001.ppt]

## Slide 1
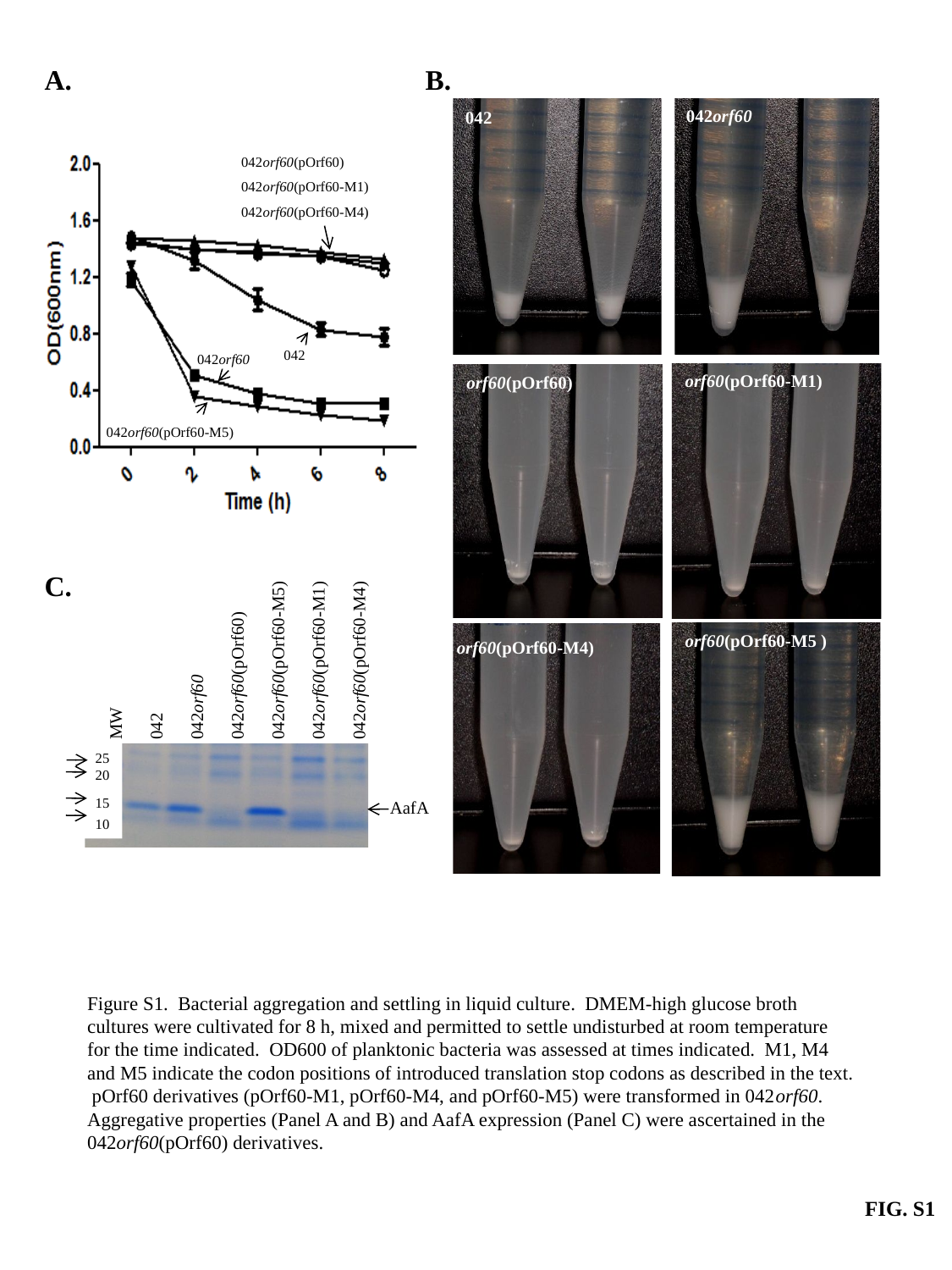

A.			B.
C.
042orf60
042
042orf60(pOrf60)
042orf60(pOrf60-M1)
042orf60(pOrf60-M4)
042
042orf60
orf60(pOrf60-M1)
orf60(pOrf60)
042orf60(pOrf60-M5)
MW
042
042orf60
042orf60(pOrf60)
042orf60(pOrf60-M5)
042orf60(pOrf60-M1)
042orf60(pOrf60-M4)
25
20
15
10
AafA
orf60(pOrf60-M5 )
 orf60(pOrf60-M4)
Figure S1. Bacterial aggregation and settling in liquid culture. DMEM-high glucose broth cultures were cultivated for 8 h, mixed and permitted to settle undisturbed at room temperature for the time indicated. OD600 of planktonic bacteria was assessed at times indicated. M1, M4 and M5 indicate the codon positions of introduced translation stop codons as described in the text. pOrf60 derivatives (pOrf60-M1, pOrf60-M4, and pOrf60-M5) were transformed in 042orf60. Aggregative properties (Panel A and B) and AafA expression (Panel C) were ascertained in the 042orf60(pOrf60) derivatives.
FIG. S1

## Slide 2
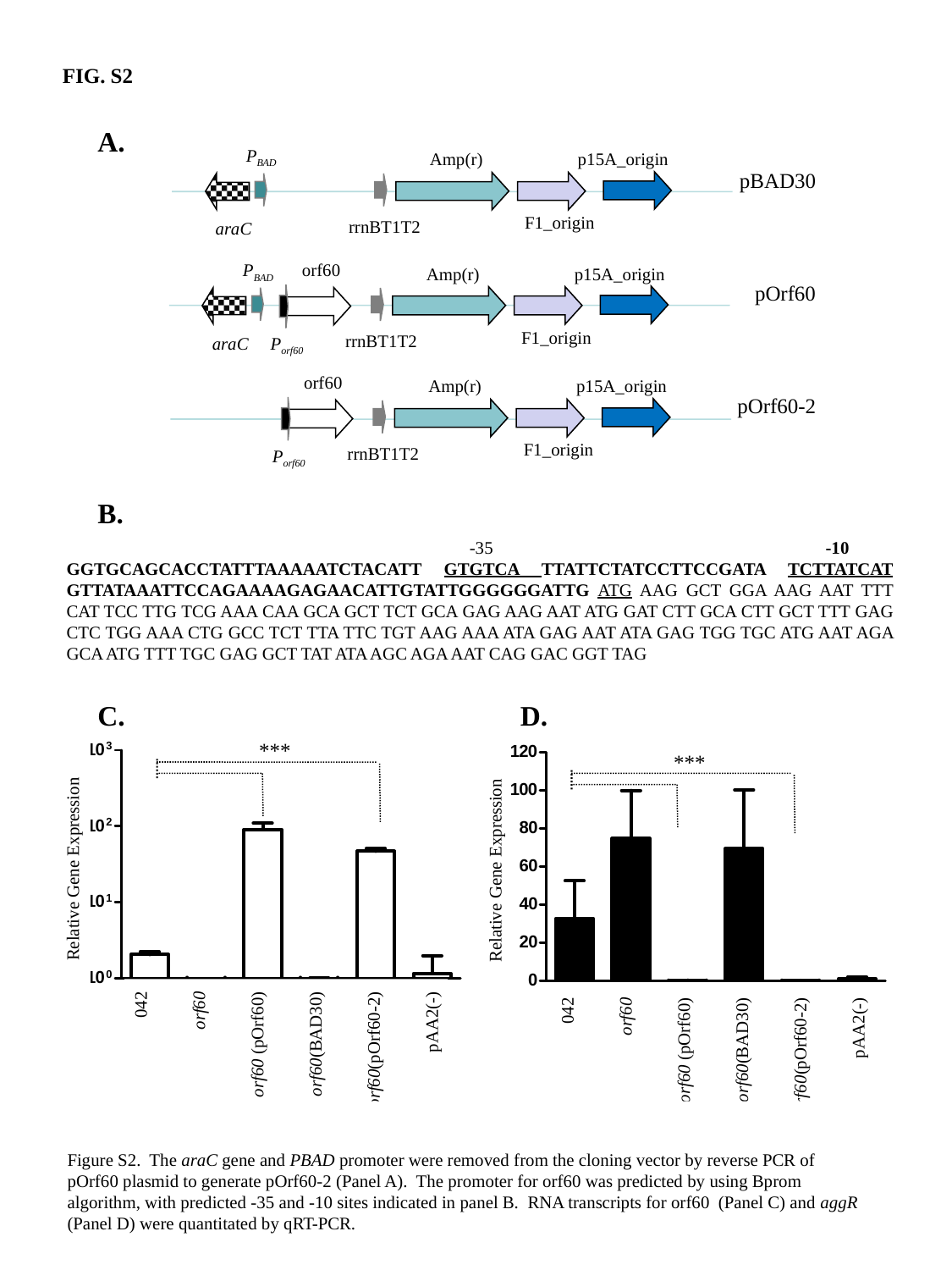

FIG. S2
A.
B.
C.		 	 D.
PBAD
p15A_origin
Amp(r)
F1_origin
rrnBT1T2
araC
pBAD30
pOrf60
pOrf60-2
orf60
PBAD
p15A_origin
Amp(r)
F1_origin
rrnBT1T2
araC
Porf60
orf60
p15A_origin
Amp(r)
F1_origin
rrnBT1T2
Porf60
			 -35		 -10
GGTGCAGCACCTATTTAAAAATCTACATT GTGTCA TTATTCTATCCTTCCGATA TCTTATCAT GTTATAAATTCCAGAAAAGAGAACATTGTATTGGGGGGATTG ATG AAG GCT GGA AAG AAT TTT CAT TCC TTG TCG AAA CAA GCA GCT TCT GCA GAG AAG AAT ATG GAT CTT GCA CTT GCT TTT GAG CTC TGG AAA CTG GCC TCT TTA TTC TGT AAG AAA ATA GAG AAT ATA GAG TGG TGC ATG AAT AGA GCA ATG TTT TGC GAG GCT TAT ATA AGC AGA AAT CAG GAC GGT TAG
***
***
Relative Gene Expression
Relative Gene Expression
 042
 orf60
 orf60 (pOrf60)
orf60(BAD30)
orf60(pOrf60-2)
pAA2(-)
 042
 orf60
 orf60 (pOrf60)
orf60(BAD30)
orf60(pOrf60-2)
pAA2(-)
Figure S2. The araC gene and PBAD promoter were removed from the cloning vector by reverse PCR of pOrf60 plasmid to generate pOrf60-2 (Panel A). The promoter for orf60 was predicted by using Bprom algorithm, with predicted -35 and -10 sites indicated in panel B. RNA transcripts for orf60 (Panel C) and aggR (Panel D) were quantitated by qRT-PCR.

## Slide 3
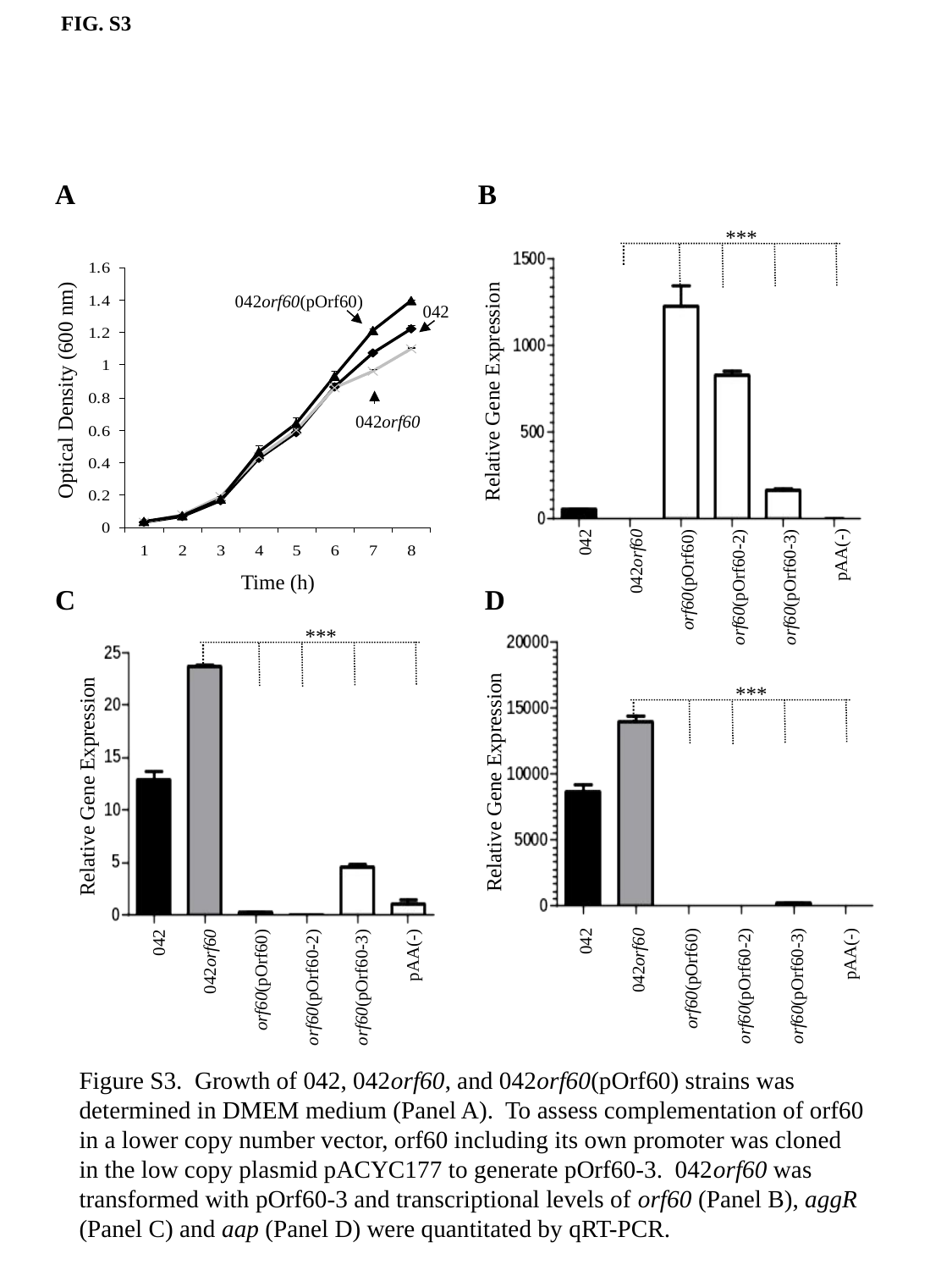

FIG. S3
A			 B
C			 D
***
042orf60(pOrf60)
042
042orf60
Optical Density (600 nm)
Relative Gene Expression
042
042orf60
orf60(pOrf60)
orf60(pOrf60-2)
orf60(pOrf60-3)
pAA(-)
Time (h)
***
***
Relative Gene Expression
Relative Gene Expression
042
042orf60
orf60(pOrf60)
orf60(pOrf60-2)
orf60(pOrf60-3)
pAA(-)
042
042orf60
orf60(pOrf60)
orf60(pOrf60-2)
orf60(pOrf60-3)
pAA(-)
Figure S3. Growth of 042, 042orf60, and 042orf60(pOrf60) strains was determined in DMEM medium (Panel A). To assess complementation of orf60 in a lower copy number vector, orf60 including its own promoter was cloned in the low copy plasmid pACYC177 to generate pOrf60-3. 042orf60 was transformed with pOrf60-3 and transcriptional levels of orf60 (Panel B), aggR (Panel C) and aap (Panel D) were quantitated by qRT-PCR.

## Slide 4
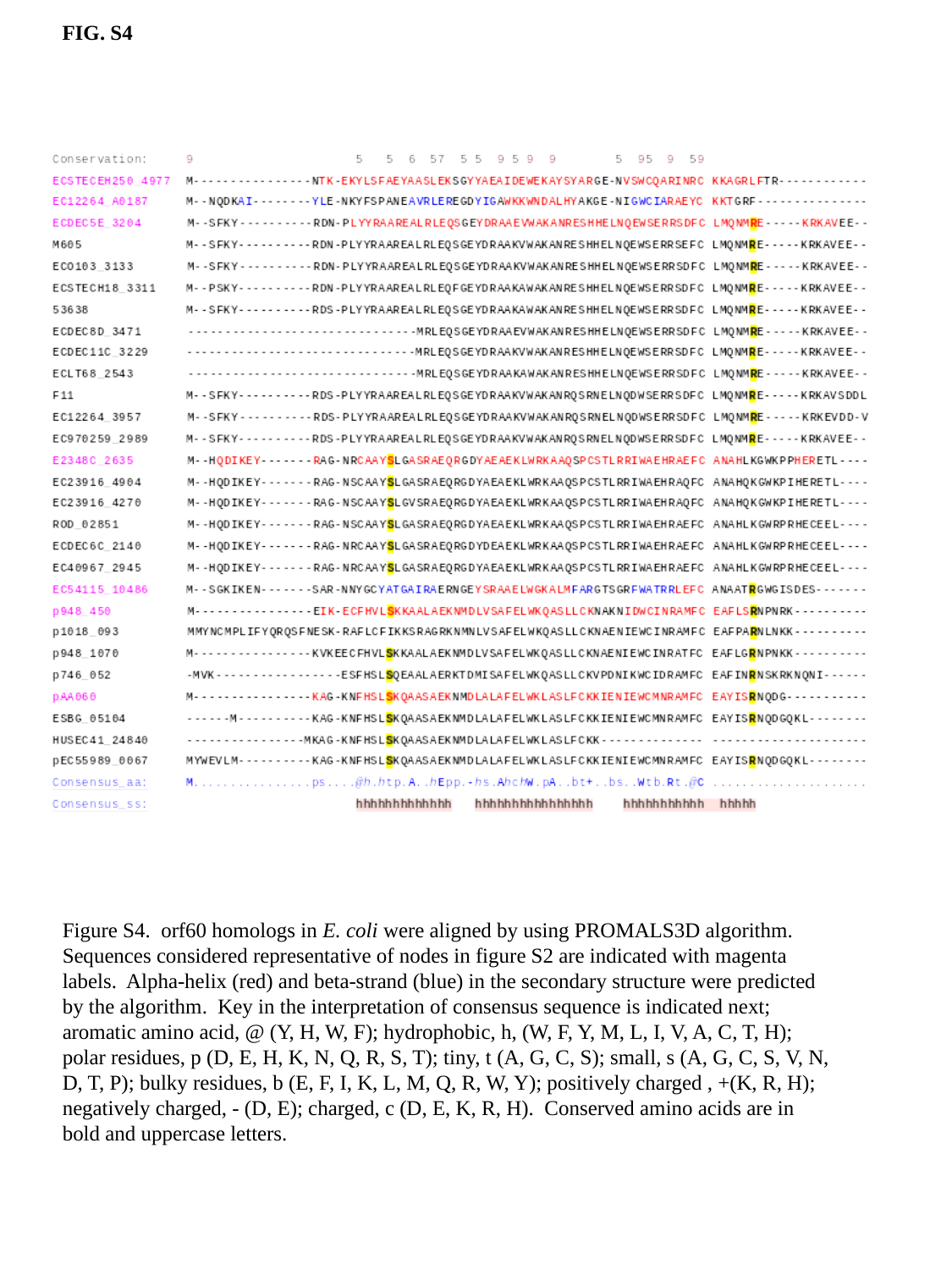

FIG. S4
Figure S4. orf60 homologs in E. coli were aligned by using PROMALS3D algorithm. Sequences considered representative of nodes in figure S2 are indicated with magenta labels. Alpha-helix (red) and beta-strand (blue) in the secondary structure were predicted by the algorithm. Key in the interpretation of consensus sequence is indicated next; aromatic amino acid, @ (Y, H, W, F); hydrophobic, h, (W, F, Y, M, L, I, V, A, C, T, H); polar residues, p (D, E, H, K, N, Q, R, S, T); tiny, t (A, G, C, S); small, s (A, G, C, S, V, N, D, T, P); bulky residues, b (E, F, I, K, L, M, Q, R, W, Y); positively charged , +(K, R, H); negatively charged, - (D, E); charged, c (D, E, K, R, H). Conserved amino acids are in bold and uppercase letters.
