# Supplementary material for: A Large Family of Antivirulence Regulators Modulates the Effects of Transcriptional Activators in Gram-negative Pathogenic Bacteria
Source: PLoS Pathog. 2014 May 29;10(5):e1004153. doi: 10.1371/journal.ppat.1004153 (PMC4038620; doi:10.1371/journal.ppat.1004153)
Supplement: Text S2 — Supporting tables. This file contains Tables S1–S3. (DOC) [file ppat.1004153.s002.doc]

| **Name**  Table S1. Strains and Plasmids | **Characteristics** | **Source** |
| --- | --- | --- |
| **Strains** | | |
| 042 | Enteroaggregative *E. coli* archetype strain 042 | 30 |
| 042*aggR* | 042 derivative carrying *aggR* deletion | 18,29 |
| 042*aggR*(pBAD*aggR*) | 042*aggR* complemented in trans by pBAD*aggR* | 18,29 |
| 042*orf60* | 042 derivative carrying *orf60* deletion | This study |
| 042*orf60*(pOrf60) | 042*orf60* complemented in trans by pOrf60 | This study |
| 042*orf60*(pOrf60-2) | 042*orf60* complemented in trans by pOrf60-2 | This study |
| 042*orf60*(pOrf60-3) | 042*orf60* complemented in trans by pOrf60-3 | This study |
| 042*orf60*(pOrf60-M1) | 042*orf60* complemented in trans by pOrf60-M1 | This study |
| 042*orf60*(pOrf60-M4) | 042*orf60* complemented in trans by pOrf60-M4 | This study |
| 042*orf60*(pOrf60-M5) | 042*orf60* complemented in trans by pOrf60-M5 | This study |
| 042*orf60*(pOrf0450) | 042*orf60* complemented in trans by pOrf0450 | This study |
| 042*orf60*(pOrf1070) | 042*orf60* complemented in trans by pOrf1070 | This study |
| 042*orf60*(pOrf02851) | 042*orf60* complemented in trans by pOrf2851 | This study |
| 042pAA2(-) | 042 derivative pAA2-plasmidless | This study |
| ETEC H10407 | Enterotoxigenic *E. coli* strain H10407 proved pathogenic in volunteers | 32 |
| H10407(pOrf0450) | H10407 transformed with pOrf0450 plasmid | This study |
| H10407(pOrf1070) | H10407 transformed with pOrf1070 | This study |
| 4820 | Mouse pathogen *C. rodentium* biotype 4280 | 33 |
| *C. rodentium orf02851* | *C. rodentium* 4820 derivative carrying orf02851 deletion | This study |
| *C. rodentium orf02851*(pOrf2851K) | *C. rodentium* *orf02851* complemented in trans by pOrf2851K | This study |
| **Plasmids** | | |
| pBAD30 | Cloning vector (Amp^r^) | Lab collection |
| pACYC177 | Cloning vector (Amp^r^) | Lab collection |
| pOrf60 | P_orf60_-orf60 from 042 strain cloned into pBAD30 (Amp^r^) | This study |
| pOrf60-2 | pOrf60-derivative without *araC* and P_BAD_ promoter | This study |
| pOrf60-3 | P_orf60_-orf60 from 042 strain cloned into pACYC177 (Amp^r^) | This study |
| pOrf60-M1 | pOrf60-derivative containing mutation in predicted codon 1 | This study |
| pOrf60-M4 | pOrf60-derivative containing mutation in predicted codon 4 | This study |
| pOrf60-M5 | pOrf60-derivative containing mutation in predicted codon 5 | This study |
| pOrf0450 | pBAD30 containing orf60 homolog from ETEC H10407 (p948_0450) | This study |
| pOrf1070 | pBAD30 containing orf60 homolog from ETEC H10407 (p948_1070) | This study |
| pOrf02851 | pBAD30 containing orf60 homolog from *C. rodentium* (ROD_02851) | This study |
| pOrf02851K | pOrf02851 containing Km^r^ marker | This study |

Table S2. Primers used for PCR

| **Name** | **DNA Sequences (5→3)** |
| --- | --- |
| Primers employed for deletion of orf60 (Region 46,236 - 46,438; GenBank FN554767.1) | |
| Orf60LRF | CTTCCAGCACGGAGCAATAGACGTAACAATCACTTAAAAAAACGGAGATAAGCCCTTTCCCATAATCTCTCAATTCCGGATTTTTCTGGTGCAGCACCTATTTAAAAA TCTACATTGTGTCATTATTCTATCCTTCCGATATCTTATCATGTTATAAATTCCAGAAAAGAGAACATTGTAGTGTAGGCTGGAGCTGCTTC |
| Orf60LRR | TTGGTGATTTTTTCTTTTTGGCGATTGATTCAGATCGCACAATCCGGGCTGAGTTCCCTCAAAGTGATCTACTATTCCGCGCAACTATTTAGACTGGTCCCCTGAAATACCAGACAGTAGATGTATCTTAAAATAAGAGATTAGGCCAGTCTAGACAGTTTTTGTTGACGACTACACTTTATGGGAATTAGCCATGGTCC |
| Primers employed for deletion of ROD_02851 (Region 319,732 - 319,967; GenBank NC_013716.1) | |
| 02851LRF | AAATTAAATTAATGCGGTGCTTTCAGTGTTTCGACTTTTACGAAATAAGGCTCGACATTAATTTCAACCACACAACCACATTTAAAATCTCTGGCTGGCGCAACAGTTTTCACCACACGACCACAACCAAACACTGCGCTGTCGGTATGTGTAAAACGAGTCCCCACCGGATATAGCTGA gtgtaggctg gagctgcttc |
| 02851LRR | CCGGGCGCGTTCTGGCCCTGCGTCGCCTCGAAATTGAGGACGAACGCTGGAATAACGCCATGACGCTGATTGGCGAAGCGGTCCAGGTGATTGGCAGCAAAAGCTATATCCGCGTGTATGAGCGCGTCGGTGATTCTGATGAATACCGCGCAATCCCGCTTGATATTGCAGGGGTTTAACATGGGAATTAGCCATGGTCC |
| Primers employed for screening of EAEC 042*orf60* and *C. rodentium* *orf02851* | |
| Orf60scF | GCCCTTTCCCATAATCTCTCAATTCC |
| Orf60scR | AATAAGAGATTAGGCCAGTCTAGACAG |
| Orf2851F | CCTTCAGGAGTAACAATAAAATCCCAGAC |
| Orf2851R | CAGACCGCGAAGTCGCTGATTGATGATTG |
| kmscF | CCGGCTGGATGATCCTCCAGCG |
| kmscR | GCCCAGTCTAGCTATCGCCATGT |
| Primers employed for deletion of hypothetical start codons M1, M4 and M5 | |
| L1F | GTTTATAAATTCCAGAAAAGAGAACATTGTATAGGGGGGATTGATGAAG |
| L1R | CTTCATCAATCCCCCCTATACAATGTTCTCTTTTCTGGAATTTATAAC |
| L4F | CATTGTATTGGGGGGATAGATGAAGGCTGGAAAGA |
| L4R | TCTTTCCAGCCTTCATCTATCCCCCCAATACAATG |
| M5F | GAACATTGTATTGGGGGGATTGTAGAAGGCTGGAAAGAATTTTCAT |
| M5R | ATGAAAATTCTTTCCAGCCTTCTACAATCCCCCCAATACAATGTTC |
| Primers employed for generation of pOrf60, pOrf0450, pOrf1070 and pOrf02581 derivatives | |
| EAECF | GAATTCGGTGCAGCACCTATTTAAAAATCTACA |
| EAECR | TCTAGACTAGTGGTGGTGGTGGTGGTGACCGTCCGTCCTGATTTCTGCTTATATAA |
| ETEC1F | CGGCTACGCCCGGGCATATGGAAATAAAAGAGTGTTTTCATGTTTTATCAA |
| ETEC1R | CGGCTACGCCCGGGTCTAGATCATTTTCTGTTTGGATTTCGGCTAAGAAAAG |
| ETEC2F | CGGCTACGCCCGGGCATATGAAAGTAAAAGAAGAGTGTTTTCATGTTCTA |
| ETEC2R | CGGCTACGCCCGGGTCTAGATCATTTTTTGTTTGGATTTCGGCCAAGAAATG |
| CrodF | CGGCTACGCCCGGGCATATGCATCAGGATATCAAAGAGTACAGAGCC |
| CrodR | CGGCTACGCCCGGGTCTAGATTAAAGTTCTTCGCATTCATGGCGTGGCC |
| Primers for qRT-PCR in ETEC genes | |
| CfaAF | GGCGGTAAAACCAGATAGCA |
| CfaAR | AACGCCTGCTCTAACATTCC |
| CfaCF | CGGTGGATTTTCGTTTGAAT |
| CfaCR | CTGCACACGTGGGTTCTTTA |
| CfaDF | GGCCATATGTTGCATTCAGA |
| CfaDR | AGCTGAGTCATCATGGCTGTT |
| CfaEF | GCCGTATCGGCAGATAAAAA |
| CfaER | CGCCATCAATTGTAGCAGTG |
| CexEF | TGGAGGCGGTAATTCTGAAC |
| CexER | GGCTACACCGATGGCATATC |
| RpoAF | TGTAGGCAATACGCTCCACA |
| RpoAR | GGTTATGTGCCGGCTTCTAC |
| Primers for qRT-PCR in *C. rodentium* genes | |
| Orf02851F | CAAAGAGTACAGAGCCGGAAA |
| Orf02851R | GTGAGCGTTAGCGCAGAACT |
| RegAF | ATTATTCATCTGCGGGATGC |
| RegAR | CGTCGCTATCAACGTGGTAA |
| KfcCF | ATGCAACCGAGACGGATAAG |
| KfcCR | ATATAGCTTGCAGCCGCACT |
| KfcEF | GAAAACCAGGACCAAAACGA |
| KfcER | CGTCAGGGCGATAAAACATT |
| KfcHF | CTTCCCATTACGATGCAGGT |
| KfcHR | CCCCAGTCAGGGTAACTTCA |
| AapF | GCATCTTCCTGGATTCCAAA |
| AapR | CGTATGAAGCAGAGCCCATT |
| AatAF | TTGGAACAGGAAGACAGCCTA |
| AatAR | TTCCCTAAACTTTCGCCAGA |
| AatBF | TGGGAAACTTTTCACCATTCA |
| AatBR | TCTCAGAGCGAAGGCTTATGA |
| AatCF | CGCAAGAATTGAGAAGGTCA |
| AatCR | CAAGAGCGAGCCTTTGTTTC |
| AatDF | TATGCTGGCTCCTTTTGCTT |
| AatDR | TCAATGGGTGAGATGGCATA |
| AatPF | GGTTATCTCCCGGCAACTCT |
| AatPR | CCCTTGCATCAAGTGAGGTT |
| RpoDF | GATCGGCGATGATGAAGATT |
| RpoDR | CGGGTAACGTCGAATTGTTT |

Table S3. Shedding of *C. rodentium* and orf02851 mutant

| Group  # mouse | Fecal Shedding (CFU/g) | | | | | | | |
| --- | --- | --- | --- | --- | --- | --- | --- | --- |
|  | Day | | | | | | | |
|  | 10 | 11 | 12 | 13 | 14 | 15 | 16 | 17 |
| *C. rodentium* |  |  |  |  |  |  |  |  |
| M1 | 4.3E+09 | 4.3E+09 | 3.0E+10 | 4.3E+09 | 1.6E+08 | 1.1E+06 | none | none |
| M2 | 1.9E+09 | 3.6E+09 | 2.4E+09 | 2.5E+09 | 1.3E+08 | 2.1E+07 | none | none |
| M3 | 1.9E+10 | 2.4E+10 | 3.3E+09 | 4.8E+09 | 2.1E+09 | 1.8E+09 | 9.7E+06 | 2.2E+08 |
| M4 | 7.6E+09 | 2.2E+10 | 6.8E+09 | dead |  |  |  |  |
| M5 | 6.8E+09 | 2.6E+10 | 3.6E+09 | 6.5E+08 | 1.8E+09 | 5.1E+08 | 1.4E+09 | 3.1E08 |
| *orf02851* |  | | | | | | | |
| M1 | 3.8E+10 | 1.6E+10 | 1.9E+10 | 7.4E+09 | 9.8E+09 | 1.0E+09 | 1.3E+07 | 4.0E+07 |
| M2 | 8.8E+09 | 1.6E+10 | 1.6E+10 | 1.1E+10 | 1.8E+10 | 1.9E+09 | 1.2E+09 | 1.6E+08 |
| M3 | 8.7E+09 | 7.3E+09 | 1.2E+10 | 1.1E+10 | 1.1E+09 | 1.0E+09 | 3.0E+7 | 3.3E+08 |
| M4 | 2.1E+10 | 6.6E+09 | 1.9E+10 | 8.8E+09 | 2.3E+10 | 4.1E+10 | 2.9E+9 | 3.5E+08 |
| M5 | 7.5E+09 | dead |  |  |  |  |  |  |
